# Supplementary material for: MaskID: An effective deep-learning-based algorithm for dense rebar counting
Source: PLoS One. 2023 Jan 26;18(1):e0271051. doi: 10.1371/journal.pone.0271051 (PMC9879489; doi:10.1371/journal.pone.0271051)
Supplement: Attachment — Submitted filename: Reply-PONE-D-21-28703.docx [file pone.0271051.s002.docx]

Dear Editors and reviewers,

Thank you very much for processing our manuscript entitled “MaskID: an effective deep-learning-based algorithm for dense rebar counting”. We have considered all the points and revised the manuscript accordingly. Enclosed please find a detailed response. For the sake of convenience, modifications are marked in red in the revised manuscript. Moreover, a clean version of revised manuscript is also re-submited.

**For Journal Requirements：**

1. Please ensure that your manuscript meets PLOS ONE's style requirements, including those for file naming. The PLOS ONE style templates can be found at https://journals.plos.org/plosone/s/file?id=wjVg/PLOSOne_formatting_sample_main_body.pdf and  <https://journals.plos.org/plosone/s/file?id=ba62/PLOSOne_formatting_sample_title_authors_affiliations.pdf>

**Reply:** Thank you very much for your informing, we have revised the manuscript so as to meet PLOS ONE’s style.

2. Thank you for stating in your Funding Statement: "This work was partially supported by the National Natural Science Foundation of China (Grant No. 61673085) by the Science Strength Promotion Program of UESTC under Grant No. Y03111023901014006."

Please provide an amended statement that declares *all* the funding or sources of support (whether external or internal to your organization) received during this study, as detailed online in our guide for authors at http://journals.plos.org/plosone/s/submit-now.  Please also include the statement “There was no additional external funding received for this study.” in your updated Funding Statement. 
Please include your amended Funding Statement within your cover letter. We will change the online submission form on your behalf.

**Reply:** We have revised the Funding Statement in the resubmission as: “"This work was partially supported by the National Natural Science Foundation of China (Grant No. 61673085), by the Science Strength Promotion Program of UESTC under Grant No. Y03111023901014006. There was no additional external funding received for this study."

3. In your Data Availability statement, you have not specified where the minimal data set underlying the results described in your manuscript can be found. PLOS defines a study's minimal data set as the underlying data used to reach the conclusions drawn in the manuscript and any additional data required to replicate the reported study findings in their entirety. All PLOS journals require that the minimal data set be made fully available. For more information about our data policy, please see http://journals.plos.org/plosone/s/data-availability.

Upon re-submitting your revised manuscript, please upload your study’s minimal underlying data set as either Supporting Information files or to a stable, public repository and include the relevant URLs, DOIs, or accession numbers within your revised cover letter. For a list of acceptable repositories, please see http://journals.plos.org/plosone/s/data-availability#loc-recommended-repositories. Any potentially identifying patient information must be fully anonymized.

Important: If there are ethical or legal restrictions to sharing your data publicly, please explain these restrictions in detail. Please see our guidelines for more information on what we consider unacceptable restrictions to publicly sharing data: http://journals.plos.org/plosone/s/data-availability#loc-unacceptable-data-access-restrictions. Note that it is not acceptable for the authors to be the sole named individuals responsible for ensuring data access.

We will update your Data Availability statement to reflect the information you provide in your cover letter.

**Reply：**Thank you very much for your suggestion. We upload minimal underlying data set as Supporting Information files in the re-submission. Some data are not uploaded to the Supporting Information since they are not suitable for fully disclosed in public. In addition, we also make a statement on data acquisition method in Data Availability statement and revised cover letter as:

“For more data acquisition, please contact corresponding author by email dbchen@uestc.edu.cn”

4. We note that the grant information you provided in the ‘Funding Information’ and ‘Financial Disclosure’ sections do not match.

When you resubmit, please ensure that you provide the correct grant numbers for the awards you received for your study in the ‘Funding Information’ section.

**Reply:** We have checked the “Funding Information” and “Financial Disclosure”. And corrected the grant information in “Funding Information” by adding another Funding “Science Strength Promotion Program of UESTC under Grant No. Y03111023901014006” such that the grant information provided in the ‘Funding Information’ and ‘Financial Disclosure’ sections is matched.

5. We note that Figure 5 in your submission contain copyrighted images. All PLOS content is published under the Creative Commons Attribution License (CC BY 4.0), which means that the manuscript, images, and Supporting Information files will be freely available online, and any third party is permitted to access, download, copy, distribute, and use these materials in any way, even commercially, with proper attribution. For more information, see our copyright guidelines: http://journals.plos.org/plosone/s/licenses-and-copyright.

We require you to either (1) present written permission from the copyright holder to publish these figures specifically under the CC BY 4.0 license, or (2) remove the figures from your submission:

a. You may seek permission from the original copyright holder of Figure 5 to publish the content specifically under the CC BY 4.0 license.

We recommend that you contact the original copyright holder with the Content Permission Form (http://journals.plos.org/plosone/s/file?id=7c09/content-permission-form.pdf) and the following text:
“I request permission for the open-access journal PLOS ONE to publish XXX under the Creative Commons Attribution License (CCAL) CC BY 4.0 (http://creativecommons.org/licenses/by/4.0/). Please be aware that this license allows unrestricted use and distribution, even commercially, by third parties. Please reply and provide explicit written permission to publish XXX under a CC BY license and complete the attached form.”

Please upload the completed Content Permission Form or other proof of granted permissions as an "Other" file with your submission.

In the figure caption of the copyrighted figure, please include the following text: “Reprinted from [ref] under a CC BY license, with permission from [name of publisher], original copyright [original copyright year].”

b. If you are unable to obtain permission from the original copyright holder to publish these figures under the CC BY 4.0 license or if the copyright holder’s requirements are incompatible with the CC BY 4.0 license, please either i) remove the figure or ii) supply a replacement figure that complies with the CC BY 4.0 license. Please check copyright information on all replacement figures and update the figure caption with source information. If applicable, please specify in the figure caption text when a figure is similar but not identical to the original image and is therefore for illustrative purposes only.

**Reply:** We are so sorry that we use a copyrighted image in Figure 5 in original manuscript. This copyrighted image is obtained by an online APP developed by Dynamic Ventures Inc. In this APP, a template should be used when count the items in an image. This APP includes free trial version and commercial version. We just use free trial version to count the rebars in image provided by us through online. According to your suggestion, we try to request permission to publish this image under the Creative Commons Attribution License (CCAL) CC BY 4.0. However, the copyright holder (Dynamic Ventures Inc ) thinks the image (Fig 5(a) in the original manuscript) can not represent the real counting result because there is no template corresponding to the original input image (Fig R1) in free trial version. The template suitable for this situation can be custom developed in commercial version. For above reason, we decide to remove the image (Fig 5(a) in original manuscript) from the revised manuscript.

Fig R1. The original image used in Fig 5 in original manuscript.

6. Please include captions for your Supporting Information files at the end of your manuscript, and update any in-text citations to match accordingly. Please see our Supporting Information guidelines for more information: http://journals.plos.org/plosone/s/supporting-information.

**Reply:** Thank your very much for your suggestion. In the original submission, the editor suggest us thoroughly copyedit the manuscript before procceding further. We ask professional scientific editing service(Editage) to thoroughly copyedit the manuscript before re-submission. So, we submit the copy of manuscript showing the changes as “Supporting Information” according to the editor’s request. It is not included in “Supporting Information” in current re-submission.

**For Reviewer #1**

Reviewer #1: The manuscript has presented a deep learning-based method for dense rebar counting. The manuscript is well eligible for publication because of the following reasons:

1. The case study and literature are well documented and drafted with a suitable English language.

2. The performance of the proposed method is accurate and outperformed the state-of-the-art methods.

Therefore, I would like to recommend the manuscript for acceptance in its present form.

**Reply:** Thank you very much for your reviewing.

**For Reviewer #2**

Reviewer #2: This paper proposes MaskID, an innovative counting method based on deep learning and heuristic strategies. First, a sliced version of the Mask region-based convolutional neural network (R-CNN) was designed to obtain the segmentation results. Their extensive experimental results demonstrated that their approach reaches a high accuracy.
The proposed system is very interesting and quite straight-forward, however, there are still many important issues that should be addressed before final publication.
1. Authors may revise the abstract to elaborate more on problem statement and their findings and contributions.

**Reply:** Thank you very much for your suggestion. We revised the abstract carefully according to your comments. Elaborated the problem, main findings and the contributions in more detail.

2. Authors may elaborate more on the novelty of their work. How it contributes to the literature. In page 2, the authors write, "The two main principles of the approach are as follows." I don't see the novelty in the MaskID algorithm used. If no one has proposed before a method like the proposed algorithm, this claim should be highlighted much more. Else, it should be indicated who has done this, and it should be indicated what the innovations of the current paper are. Furthermore, briefly describe the major contributions in bullet form, just before the organization paragraph.

**Reply:** Thanks for your comments. In this paper, we have improved an original Mask RCNN [R1] by slicing and rescaling so as to capture more details in a large-scale image for counting rebars accurately under ultra width or ultra high stacking. Moreover, according to the characteristics of rebar stacking, we have designed intelligent denoising strategies to remove the noise in the results obtained by improved Mask RCNN so that counting results are improved further. We gave more details about maskID algorithm and explained it clearly according to your suggestion. Furthermore, we listed major contributions before organization paragraph in the revised manuscript, that is:

The key contributions of the paper are as follows:

- A novelty algorithm maskID is proposed to count the rebars in complex environment.
- An improved version of Mask RCNN is presented through slicing and rescaling so as to capture more details in a large-scale image.
- Intelligent denoising strategies are designed to remove the noise in the results obtained by improved Mask RCNN.
- Experiments on open-competition and test-platform datasets show that maskID can obtain more accurate counting results than other state-of-the-art algorithms.

[R1] He K, Gkioxari G, Dollar P, G. Ross. Mask RCNN. In: The IEEE International Conference on Computer Vision; 2017. P. 2980-2988.

3. Introduction can be improved by having four clear and concise subsections on motivation of your research; background and related works; list of key contributions; and organization of the paper.

**Reply:** Thank you very much for your constructive suggestions. We have improved the introduction througth four subsections, including:

Motivation

Background and related works

Key contributions

Organization of the paper

4. A comparative literature review may require identifying the problem/ research gap in Section "Materials and Methods". A few of the references are missing some information, may complete them critically. Provided references are better enough. However, authors are recommended to consider more latest and related such as,
https://www.sciencedirect.com/science/article/abs/pii/S1047320321001711
https://www.sciencedirect.com/science/article/abs/pii/S1389128619306978
<https://ieeexplore.ieee.org/abstract/document/8606255>.

**Reply:**

We have given comparative literature review in the revised manuscript to show the research gap on identifying or counting problem. More latest and related references [R2, R3, R4] as well as their descriptions are also added in the Section “Introduction” and Section "Materials and Methods" of revised manuscript.

# [R2] Wu Y, Liu W, Wan S. Multiple attention encoded cascade R-CNN for scene text detection. Journal of Visual Communication and Image Representation. 2021; 80: 103261.

[R3] Wan S, Goudos S. Faster R-CNN for multi-class fruit detection using a robotic vision system. Computer Networks. 2020; 168:107036.

[R4] Zhao Y, Li H, Wan S, Sekuboyina A, Hu X, Tetteh G, et al. Knowledge-Aided Convolutional Neural Network for Small Organ Segmentation. IEEE Journal of Biomedical and Health Informatics. 2019; 23(4):1363-1373.

5. What are the shortcomings of the algorithm and what is the room for improvement? I suggest adding a brief description in the conclusion section.

**Reply:** Thank you for your suggestions. Actrully, fixed slicing and overlapping size are set in the current method. However, a rebar with certain real size might has different image size in different scence. So, the identifying results might not stabilized if the image size of rebar is particularly small or large. For example, the image size of rebar is larger than 400 × 400 pixels or smaller than 5 × 5 pixels, it is difficult to identify the rebars accurately utilizing the proposed algorithm with sliced window 384 × 384 pixels. In order to further improve the generalization ability of the algorithm, several slicing and overlapping size might be used to identify the rebars, and the best result will be adopted. Brief descriptions about shortcommings of the proposed algorithm and the improvement in the future are added in the summary of revised manuscript according to your suggestion.
