# Supplementary material for: MaskID: An effective deep-learning-based algorithm for dense rebar counting
Source: PLoS One. 2023 Jan 26;18(1):e0271051. doi: 10.1371/journal.pone.0271051 (PMC9879489; doi:10.1371/journal.pone.0271051)
Supplement: Attachment — Submitted filename: response-2nd.docx [file pone.0271051.s003.docx]

Dear Editor,

Thank you very much for processing our manuscript entitled “MaskID: an effective deep-learning-based algorithm for dense rebar counting”. We have considered all the points and revised the manuscript accordingly. Enclosed please find a detailed response. For the sake of convenience, modifications are marked in red in the revised manuscript. Moreover, a clean version of revised manuscript is also re-submited.

1) Section- Datasets: As mentioned by the authors 2 datasets have been used, it will be advisable if sample images from the 2 datasets should be shown to get an overview of the type of images. The first dataset has 200 images, but for the 2nd dataset, it is not clear, how many images are there. The results are only tabulated in the form of Table 2, using F1 score. The division of the datasets into training/validation and testing is not given. Nor the training and testing accuracy is mentioned. These parameters should be mentioned in the manuscript.

**Reply**: We are so sorry that we don’t describe the datasets clearly. There are total 450 images in the first dataset provided by DataFountain competition with 250 images being training set and 200 images being testing set. The division of the first dataset in MaskID is same as that in benchmark methods. The results in Table 2 (Table R1 in the reply) are averaged on 200 testing images of the first dataset. Besides, average values of Precision and Recall on 200 testing images of the first dataset are also added in Table 2 in the revised manuscript. Because the results of benchmark methods are directly taken from the references, the precision and recall of these methods are not listed in Table 2 since these two metrics were not given in the original references.

Table R1 The average results on 200 test images of the first dataset.

In our study, we just use 250 images of the first dataset from DataFountain competition to train MaskID. For the second dataset, just one image with 6405*3597 pixels obtained from our own experimental platform to evaluate the performance of MaskID on large scale image. Precision, Recall and F1 score are added in the revision of manuscript so as to clarify the result clearly, as shown in Table R2 (Table 3 in the revised manuscript).

Table R2 The rebar counting results on dataset 2

| The number of real rebars | Number of recognition | Number of missing identification | Number of misidentification | Precision | Recall | F1-Score |
| --- | --- | --- | --- | --- | --- | --- |
| 7453 | 7402 | 67 | 16 | 0.9978 | 0.9910 | 0.9944 |

2) it looks like the combined results from the 2 datasets are given, which means that the 2 datasets were combined for the analysis, or dataset 2 was only used for testing and dataset 1 for training? Need to explain

**Reply**: We are so sorry for confusing description. The model MaskID is trained on 250 images of dataset 1. The rest 200 images of dataset 1 and dataset 2 are used for testing. The results of dataset 1 is listed in Table 2 (Table R1 in the reply) in the revised manuscript. And the results of dataset 2 are shown in Fig 5 and Table 3 (Table R2 in the reply) in the revised manuscript. Actually, besides the results of large scale image given in manuscript, we have tested some other real scenarios recently, the results are rather good, as shown in Fig R1 and Table R3 as an example.

Fig R1 Recognition results on real scenario

Table R3 The rebar counting results on real scenario

| Real number of rebars | Number of recognition | Number of missing identification | Number of misidentification | Precision | Recall | F1 score |
| --- | --- | --- | --- | --- | --- | --- |
| 980 | 980 | 5 | 5 | 0.9949 | 0.9949 | 0.9949 |

3) In addition to the F-1 score it is advisable to include the values for precision and recall.

**Reply**: Thank you very much for your constructive suggestions. The precision and recall of our model is added in Tables 2 and 3 in the revised manuscript. Because the results of benchmark methods are directly taken from the references, the precision and recall of these methods are not listed in Table 2 since these two metrics were not given in the original references.

4) The details of the model should also be included.

### Reply: Thank you very much for your constructive suggestions. The proposed model includes two main parts. The first is improved mask RCNN, and the second is noise elimination. To better understanding improved Mask RCNN, several details are elaborated. Firstly, for model architecture, Mask RCNN with ResNeXt101[R1] backbone with four stages is adopted and the backbone is refined by replacing standard convolutional layers with deformable convolutional layers in last three stages. Besides, in the first stage, multiple candidate bounding boxes are proposed to classify foreground and background and regress bounding-box coordinate offsets. In the second stage, features are extracted using ROIAlign from each candidate box and performs classification and bounding-box regression in parallel. Secondly, for data processing, in order to effectively detect multiscale rebars in a large resolution image, the image is split into several small scale images and each one is then rescaled into 1024*1024 pixels. Finally, the model is trained by using sgd optimizer with 0.02 learning rate.

[R1] Mahajan D, Girshick R, Ramanathan V, He K, Paluri M, Li Y, et al. Exploring the limits of weakly supervised pretraining. In: European Conference on Computer Vision; 2018. p. 185–201.

5) Table 2 compares the result of the proposed algorithm with the state of the art. Are all these methods use the same datasets?

**Reply**: We are so sorry for unclear description. All methods listed in Table 2 use the same dataset. Actually, Table 2 shows the results of methods on dataset 1. The F1 scores are averaged on 200 images of dataset 1. We have revised the description in the revision of manuscript so as to eliminate ambiguity.

6) The references are a bit confusing, Ref 9 and Ref 21 are the same. It is mentioned as Retina Net, in which a dense detector is designed and named as retina NET. IT is not clear if the same dataset is used, then how to justify the results in Table 2.-yes

**Reply**: We are so sorry for confusing. Ref 9 and Ref 21 are duplicated in the first revision of manuscript for our careless. We have removed Ref 21 from the first revision of manuscript.

7) Similar to above, ref 4 and Ref 22 are the same.

**Reply**: We are so sorry for confusing. Ref 4 and Ref 22 are duplicated in the first revision of manuscript for our careless. We have removed Ref 22 from the first revision of manuscript.

8) for table 2, if the same dataset is not used then the comparisons are not meaningful, it will be better if the authors give a detailed table in which they include the type of dataset the no of images, and results to get an overview of what is happening.

**Reply**: Table 2 is the results of dataset 1. All methods listed in Table 2 use the same dataset, i.e., 200 testing images of dataset 1. The F1 scores in Table 2 are averaged on 200 testing images of dataset 1. We have revised the corresponding descriptions in the revision of manuscript so as to describe Table 2 clearly.

9)There are some spelling and grammatical mistakes that should be corrected. For example, line 33, should be scene.... line 85 novelty----novel.

Reply: Thank you very much for pointing out these problems. We have checked the manuscript thoroughly and corrected the spelling and grammatical mistakes.
